# The Baffling Diagnosis of a Rare Case of Polypoid Endometriosis: Neoplasm Mimicking in a Young Pregnant Woman—A Case Report and Literature Review

**DOI:** 10.3390/diagnostics15192460

**Published:** 2025-09-26

**Authors:** Nicolae Gică, Ioana-Stefania Bostan, George-Dumitru Gheoca, Raluca Chirculescu, Alexandru-Gabriel Bran, Anca Maria Panaitescu, Claudia Mehedințu

**Affiliations:** 1 Faculty of Medicine, “Carol Davila” University of Medicine and Pharmacy, 050474 Bucharest, Romania; gica.nicolae@gmail.com (N.G.); george.gheoca94@gmail.com (G.-D.G.); ralucachirculescu@gmail.com (R.C.); anca.panaitescu@umfcd.ro (A.M.P.); claudia.mehedintu@umfcd.ro (C.M.); 2 Filantropia Clinical Hospital, 011132 Bucharest, Romania; 3 Department of Radiology, University Emergency Hospital Bucharest, 050098 Bucharest, Romania; alexbran2018@gmail.com

**Keywords:** polypoid endometriosis, pregnancy, diagnosis, malignancy, imaging, treatment, surgical intervention

## Abstract

**Background**: Polypoid endometriosis is a rare variant of endometriosis that presents as a tumorous mass, making it difficult to differentiate it from a malignant tumor. It usually occurs in perimenopausal women or those undergoing hormone therapy, and its presence in a young pregnant woman is extremely uncommon. **Case Presentation**: This article describes a rare instance of polypoid ovarian endometriosis in a pregnant woman, a condition with few documented cases in the medical literature. An adnexal mass was discovered incidentally during a routine prenatal ultrasound, with imaging features that raised the suspicion of a neoplastic process and prompted surgery to exclude malignancy. However, histopathological examination of the excised lesion confirmed features compatible with polypoid endometriosis, without revealing evidence of cancer. This case highlights the diagnostic challenges of differentiating polypoid endometriosis from ovarian neoplasms, especially during gestation, where imaging findings can be ambiguous. In addition, the hormonal environment inherent in pregnancy may exacerbate the proliferative behavior of endometriotic lesions, thus complicating clinical evaluations. The presence of an adnexal mass in such a setting often requires careful evaluation to balance the risks of surgery with the potential consequences of delayed diagnosis. The data presented emphasize the importance of an accurate diagnosis. In conclusion, a well-coordinated approach ensures the protection of maternal and fetal health. **Conclusions**: By prioritizing accurate diagnosis and personalized treatment plans, physicians can minimize complications and improve outcomes for both mother and child.

## 1. Introduction

Polypoid endometriosis is an uncommon variant of endometriosis that is characterized by the development of polypoid masses originating from ovarian or extra-ovarian endometriotic lesions [1,2]. This condition is benign and exhibits histological features that resemble those of endometrial polyps. However, it can present significant challenges in distinguishing it from malignancy, both in the preoperative setting and during surgical intervention. The literature is limited—a few dozen reported cases and imaging studies. Extensive research is lacking, and diagnostic and treatment strategies are based on case reports and small series [3,4]. Polypoid endometriosis is a rare and benign condition, typically found in post-menopausal women [5,6,7]. Its histological features are similar to those of endometrial polyps or typical endometriosis, but it exhibits exophytic growth [8,9,10]. This type of endometriosis can occur in various locations within the body, including the ovaries, uterine serosa, cervical canal, vaginal mucosa, ureters, fallopian tubes, great omentum, colon, bladder, peritoneum, and the paraurethral and paravaginal regions. It is characterized by rapid growth [11,12]. The clinical presentation and imaging findings of polypoid endometriosis can be misleading, as it may easily resemble malignancy. This can lead to medical dilemmas and challenges in establishing a diagnosis, both before and sometimes even during surgery [13]. Polypoid endometriosis is an uncommon and noteworthy variant of endometriosis, distinguished by the formation of polypoid lesions that can mimic clinically and histologically malignancies [14,15].

The medical management of polypoid endometriosis primarily involves hormonal therapies, including GnRH agonists and antagonists, which are utilized to suppress estrogen production. This therapeutic approach aims to reduce endometrial proliferation and alleviate associated symptoms. Furthermore, the incorporation of add-back therapy utilizing low-dose progestins or estrogens may help mitigate the side effects of such treatments [16,17]. Available treatment modalities encompass oral, injectable or intrauterine methods, such as the levonorgestrel-releasing intrauterine system (LNG-IUS), all designed to diminish endometrial activity and inflammation. In refractory cases, aromatase inhibitors may be considered to inhibit estrogen production at both peripheral and tissue levels. For the management of pelvic pain and dysmenorrhea, non-steroidal anti-inflammatory drugs (NSAIDs) serve as an effective option [18,19]. Surgical intervention may be indicated in situations where symptomatic lesions fail to respond to medical therapy, particularly when substantial masses are identified or there is suspicion of malignancy. It is critical to conduct pre-surgical counseling to ensure that patients are informed about the potential implications of surgery, as well as the associated risks [20,21]. The overall treatment strategy for polypoid endometriosis typically comprises a combination of medical therapy (to address symptoms and prevent recurrence), surgical intervention (to excise lesions and confirm the diagnosis) and fertility-focused care, if appropriate. This comprehensive approach is tailored to the individual needs of each patient, ensuring an effective and holistic management plan [22].

The difficulty in differentiating this condition is particularly pronounced during pregnancy, as the clinical and radiological presentations of hormonally responsive tissues frequently overlap with those associated with neoplastic growth [23,24]. Several compelling aspects supported the choice of the subject of this study.

Our analysis focused on a noteworthy case from our hospital involving a pregnant woman diagnosed with polypoid endometriosis that presented neoplastic features. A comprehensive search of the specialized literature revealed that such cases are quite rare, which underscores the significant challenges associated with diagnosing polypoid endometriosis due to its tendency to mimic neoplastic conditions.

This review highlights the gap in the existing literature regarding polypoid endometriosis in pregnant women. Although it begins with the presentation of a rare case, the article supports and correlates with findings from two recent studies [25]. It emphasizes the challenges in diagnosing endometriotic lesions during pregnancy and exploring potential treatment options. The data presented aligns with existing literature and aims to enhance understanding of this condition while encouraging further research in the field.

This analysis has great clinical significance because it can contribute to understanding the pathology of polypoid endometriosis and ensure better management of the patient’s condition, since such cases may involve unique challenges or complications of the therapeutic approach. Furthermore, the examination of such cases underscores the complexities and intricacies involved in differential diagnosis. It is known that during pregnancy, the endometrium thickens, and the uterus enlarges, making it more difficult to distinguish polypoid endometriosis from hyperplastic endometrium or malignancy. It fosters interdisciplinary collaboration by engaging specialists from diverse fields, including gynecology, pathology, radiology, and oncology. Also, the study has an educational value by providing the opportunity to inform health professionals and researchers about the atypical presentations of polypoid endometriosis, promoting awareness and early diagnosis.

## 2. Case Report

### 2.1. Case Overview

This paper presents the case of a young pregnant woman who, during a routine ultrasound, had an incidental finding of an ovarian mass, which was initially misdiagnosed as cancer but was subsequently re-evaluated and accurately diagnosed as polypoid endometriosis. A 37-year-old nulliparous patient, 13 weeks and 4 days pregnant, presented to the maternal–fetal medicine specialist for a first-trimester morphology at Filantropia Clinical Hospital, Bucharest, Romania. In pregnant women, distinguishing between polypoid ovarian endometriosis and neoplastic growths presents significant challenges. These difficulties arise from various factors, including hormonal stimulation that alters the ovarian environment, limitations in imaging techniques that may not provide clear differentiation, overlapping clinical features that can obscure a definitive diagnosis, and the restricted options for biopsy due to the delicate nature of pregnancy. In order to effectively address these complexities, it is imperative to implement a comprehensive and multidisciplinary approach. This approach includes the judicious use of advanced imaging modalities, vigilant monitoring of the patient’s condition, and, when warranted, selective surgical intervention. Such a coordinated effort is necessary to ensure accurate diagnosis and effective management of these conditions during pregnancy. Written informed consent was obtained from the patient for the publication of this case and any accompanying images.

### 2.2. Patient History

#### 2.2.1. Personal History

The patient reported no significant personal history. Menstrual cycles were regular and not accompanied by excessive pelvic pain or bleeding. There were no fertility issues implicated, and the current pregnancy, which was also her first, was obtained spontaneously without any additional effort. Regarding previous surgeries, only an appendectomy was reported, but it was many years back, during childhood. No signs or symptoms suggestive for endometriosis, such as dysmenorrhea, dyspareunia, or chronic pelvic pain, were mentioned.

#### 2.2.2. Family History

The patient had no significant family history.

### 2.3. Investigations

#### 2.3.1. Laboratory Examinations

The peripheral blood tests conducted on the patient yielded results within normal limits. Additionally, the assessment of tumor markers, including CA-125, HE4, and CA 19-9, revealed values consistent with the normal range: CA-125 measured at 17.3 U/mL, HE4 at 45.5 pmol/L, and CA 19-9 registered as less than 2.

#### 2.3.2. Physical and Ultrasound Examination

**Figure 1:**
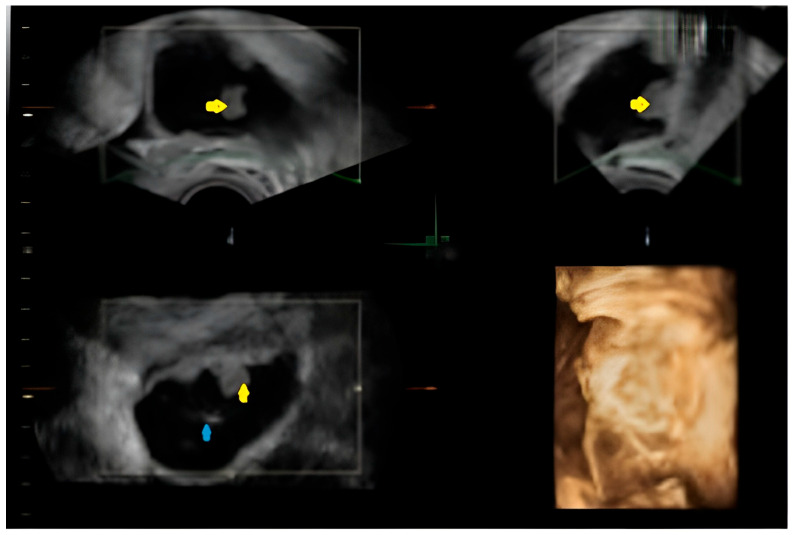
Transvaginal pelvic ultrasound—2D mode (top left, top right, bottom left) shows the right adnexal mass and its mixed components (the yellow arrows indicate internal vegetations, while the blue one points to intra-cystic fine septation) and 3D volume rendering (bottom right) allowing assessment of the lesion in a spatial manner.

The patient had an overall good health condition. Abdominal distension was appropriate for gestational age and she reported no unusual pain, bleeding or amniotic fluid loss. The first trimester morphology showed as follows: adequate growth for gestational age, without detectable structural defects; on the right ovary—a formation suggestive of a complex ovarian cyst measuring 4.98/3.75 cm, with a mixed non-echogenic and solid component; left ovary of normal appearance (Figure 1).

It is worth mentioning that on the pregnancy confirmation ultrasound, the patient did not present any structural abnormality at the level of the adnexa and that the patient had absolutely no clinical signs or symptoms suggestive of an ovarian mass.

**Figure 2:**
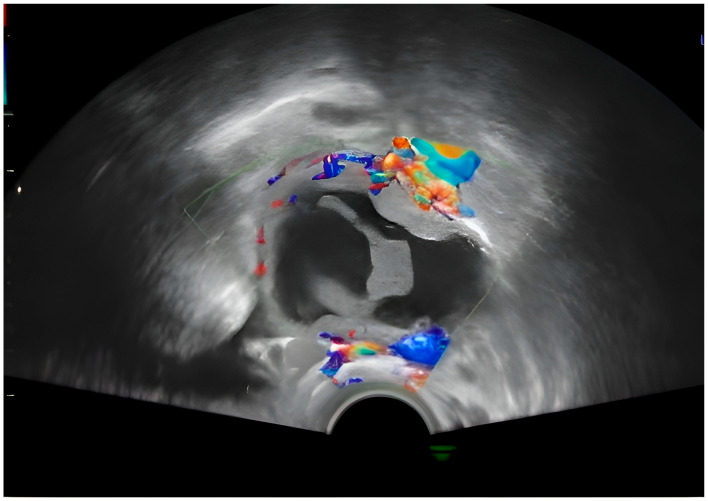
Transvaginal pelvic ultrasound with Doppler examination showing (seen as blue and red) a right adnexal growth with a mixed hypo-hyperechoic component and the presence of a peripheral Doppler signal, suggestive of a complex mass lesion.

Approximately 3 weeks later, the patient performed a routine check-up with the attending physician, during which the following aspects were detected by ultrasound: Pregnancy 16 weeks and 2 days in progress; on the right laterouterine site an image of about 5 cm is visualized, with regular contour, mixed content, multiple intracystic vegetations, of which the largest was 3.4/3.2 cm, with Doppler signal present and several small vegetations of approximately 0.2 cm; on the left laterouterine site—an image of 3/1.8 cm, with regular contour, intracystic vegetation and Doppler signal; no free intraperitoneal fluid was detected. Following this ultrasound, the suspicion of ovarian neoplasm was raised and the patient was advised to undergo more advanced imaging investigations (Figure 2).

A single, well-defined, predominantly anechoic cystic structure is seen in the right adnexal region. The lesion is thin-walled, with no evidence of papillary projections or solid mural nodules. Possible fine internal septation or debris layering may be present (as seen in the coronal and transverse 2D views), suggesting a complex cyst, although the overall appearance remains largely cystic. The 3D reconstruction provides a spatial view of the lesion, showing a rounded, fluid-filled structure. No calcifications or highly irregular wall thickening are visible (Figure 1).

The central structure appears anechoic to hypoechoic, indicating that it is filled with fluid. The internal appearance of the cyst seems to have some irregularity or septation, which may suggest a complex cyst. The red and blue signals in the walls or septa show vascularization. This Doppler scan helps to assess blood flow. In this image, peripheral flow is visible (Figure 2).

**Figure 3:**
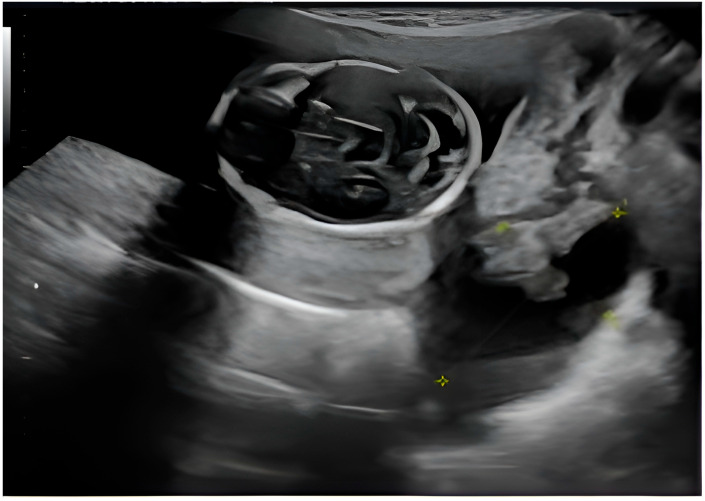
2nd trimester ultrasound depicting the fetal cranium (upper left) and a structurally modified right adnexa (lower right) which does not usually appear during routine obstetric evaluation.

The presence of ovarian structures during fetal ultrasound suggests the possibility of the existence of a pelvic mass or ovarian pathology, possibly in the presence of pregnancy or as part of a broader pelvic affliction. A well-circumscribed cystic lesion with internal components—likely a complex ovarian cyst—can be seen. The cyst appears to have thin septa or echogenic areas. There is no clear visible solid nodular area. Fetal anatomy—in the upper left area of the image, there appears to be a rounded structure resembling a fetal head, with developing cranial structures and brain anatomy (Figure 3).

The yellow cross indicates measurements taken, likely of cyst diameter or internal features, which help classify size and complexity, thus influencing management decisions (Figure 3).

#### 2.3.3. MRI Findings

**Figure 4:**
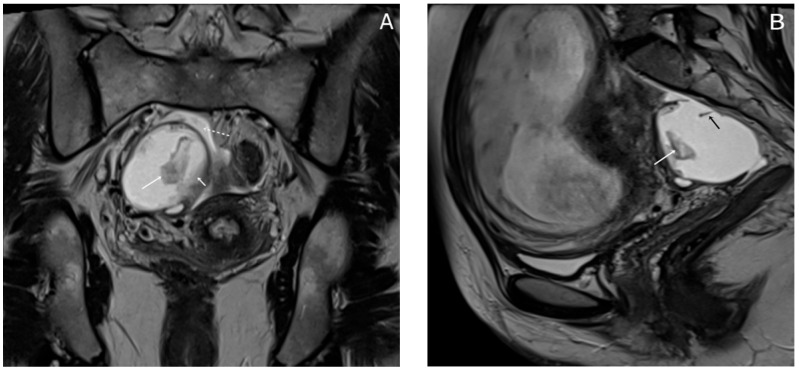
Sagittal TSE-T2WI images (**A**) and coronal TSE-T2WI (**B**) images show a thin smooth-walled unilocular cystic mass located behind the uterus, measuring (56 × 45 × 60 mm) with irregular soft tissue components (short arrow), papillary projections protruding into the lumen (long arrow) and incomplete internal septations (black arrow); there is a small amount of fluid around the lesion (dotted arrow).

**Figure 5:**
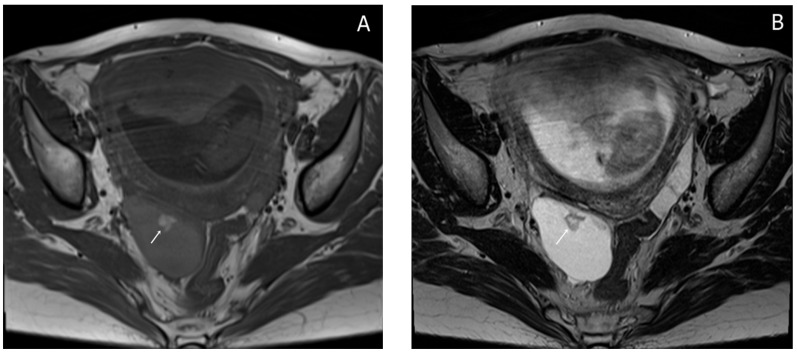
Axial T1WI-TSE image (**A**) and axial T2WI-TSE image (**B**) show a unilocular right ovarian cystic mass. The signal intensity of the cystic component demonstrates intermediate signal on T1WI and high signal on T2WI. The intracystic papillary projection appears hyperintense on T1WI and hypointense on T2WI (arrows).

**Figure 6:**
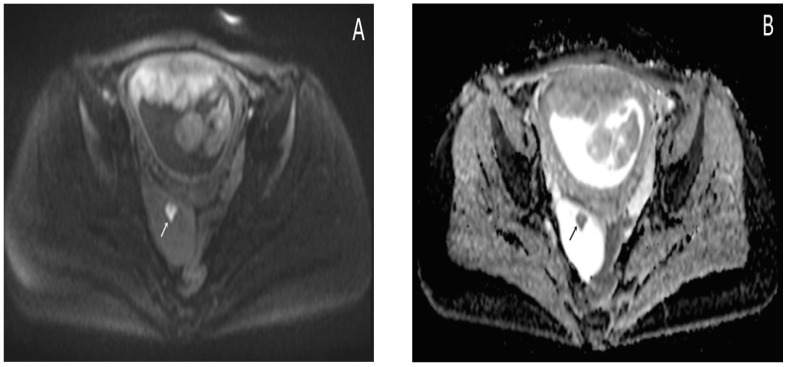
Axial DWI b800 s/mm^2^ image. (**A**) hyperintensity of the papillary projection of the endometrioma (arrow); apparent diffusion coefficient (ADC) map axial image. (**B**) low signal intensity of the papillary projection (arrow); these findings show restricted diffusion of the papillary projection; the cystic component shows no restricted diffusion.

Almost a week later she performed a native MRI which highlighted: uterus with cranio-caudal dimensions of approximately 17 cm, no suspicious lesions at the level of the fetal content are visualized on the sequences performed; the left ovary measures approximately 37 mm in the anteroposterior plane; a space-replacing cystic process (ORADS4), located at the level of the right ovary, extended over an area of up to 56/45/60 mm (ap/ll/cc), reveals filiform septation of up to 2 mm in the right anterolateral quadrant, inhomogeneous solid inclusions with a thickness of up to 9 mm, approximately 3 papillary projections, intralesional hemorrhagic stigmata and a wall with a thickness of up to 3 mm; no space-replacing processes evident on MRI at the level of the rectal wall; no bone lesions with an oncological meaning evident in the examined MRI field; infracentimetric lymph nodes in the short axis located at the level of the internal and external iliac stations bilaterally (Figure 4, Figure 5 and Figure 6).

Considering all the investigations performed, the need for surgical intervention was decided in order to establish the diagnosis with certainty and the subsequent therapeutic conduct.

## 3. Surgical Intervention and Histopathological Evaluation

**Figure 7:**
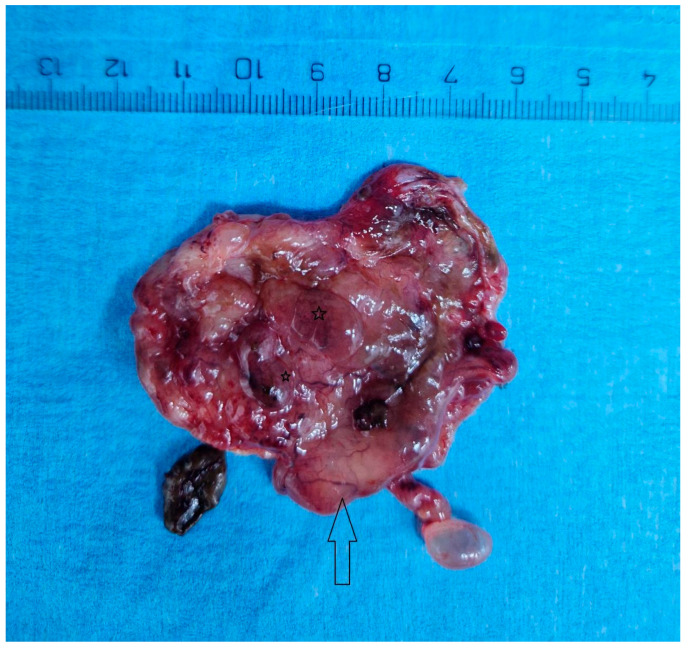
Ovarian cyst displaying an internal surface transitioning from a shade of gray to pink, characterized by polypoid projections (stars) and prominently visible vascular structures (arrow).

On 20 November 2024, at 19 weeks and 5 days of gestation, under spinal anesthesia, surgery was performed. Upon entering the peritoneal cavity, the following aspects were observed: minimal peritoneal free fluid—which was sent for cytology diagnosis and came back negative for malignancy (smear consisting of frequent red blood cells, denuded nuclei and isolated and focal mesothelial cells grouped in plaques); pregnant uterus enlarged in volume as a 20-week pregnancy; left adnexa of normal macroscopic appearance; right adnexa adherent to the posterior uterine wall—digital adhesiolysis was performed—right fallopian tube of normal macroscopic appearance and a multiloculated cystic ovary of approximately 6/5 cm; the rest of the intra-abdominal viscera seemed macroscopically normal, within the limits of exploration, given the delicate nature of pregnancy (Figure 7).

Thus, a right oophorectomy was performed, with the extracted specimen being sent for histopathological examination.

**Figure 8:**
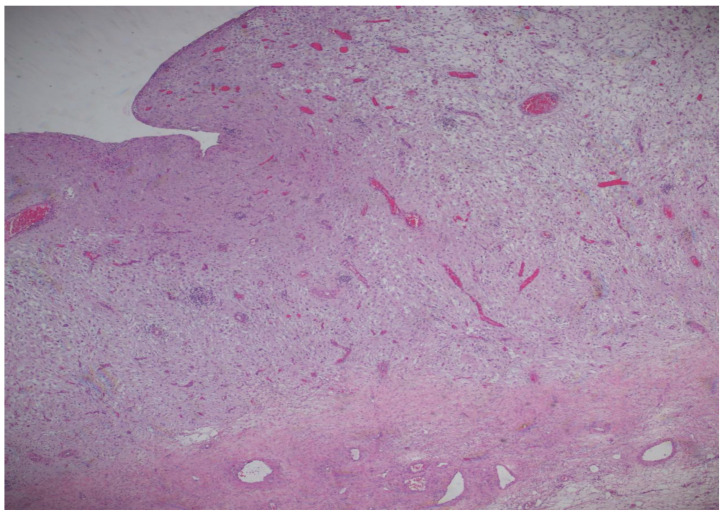
Microscopic image stained by hematoxylin–eosin at 100× magnification. The cyst exhibits a polypoid architecture, characterized by polygonal cells with a decidual appearance, and a delicate capillary network diffusely distributed.

**Figure 9:**
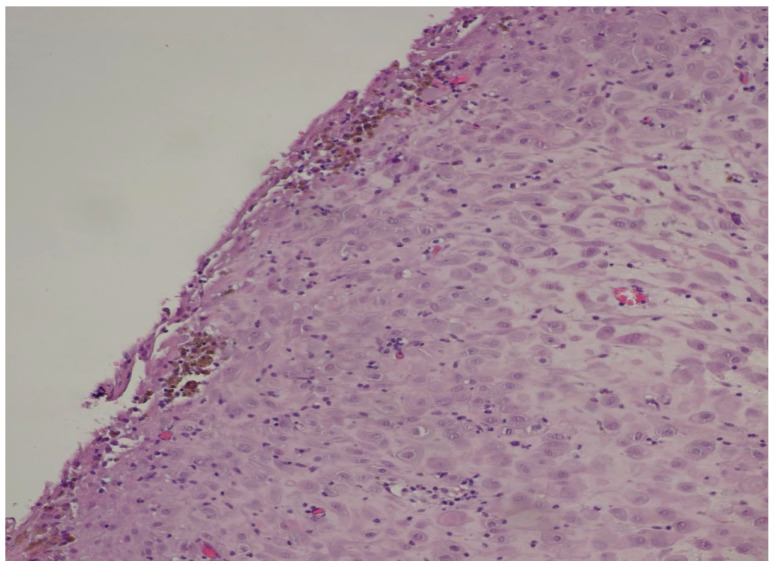
Microscopic image stained by hematoxylin–eosin, at magnification 200×.

The histopathological result came as follows: (1) Polypoid ovarian endometriosis, pregnancy-induced changes. (2) Right ovarian serous cystadenoma. ICD-O: 8441/0. 3. Peritoneal fluid and benign cytology (Figure 8 and Figure 9).

The microscopic examination reveals a decidualized stroma exhibiting rare capillary formations, small lymphocytes, and small clusters of hemosiderin-laden macrophages (serving as an indirect indicator of endometriosis). Notably, there is an absence of endometrial-type glands within the stromal tissue.

## 4. Post-Operatory Follow-Up

The surgical procedure was carried out without any intraoperative complications and postoperative recovery was uneventful. The patient remained hemodynamically stable, with no signs of infection, excessive bleeding or thromboembolic events and was later on discharged with appropriate analgesia (in order to prevent pre-term labor) and postoperative care instructions.

Following surgery, the patient continued routine obstetric follow-up, being closely monitored by an extremely attentive team comprising a maternal–fetal specialist, her usual Ob-Gyn and the surgeons who carried out the procedure. Serial ultrasounds demonstrated normal fetal growth and development, with no evidence of intrauterine growth restriction or placental abnormalities. The patient reported no recurrent pelvic pain, vaginal bleeding or signs of obstetric complications and all laboratory investigations remained within normal range. Throughout the remainder of the pregnancy, the patient maintained excellent overall health, adhering to recommended prenatal care and nutritional guidelines.

At 39 weeks, the patient had an uneventful elective cesarean section, resulting in the birth of a healthy female neonate weighing 3550 g, with Apgar scores of 9&10. The newborn showed no congenital anomalies and the postpartum course was uncomplicated for both mother and child.

Before discharge, the patient was informed about the risk of recurrence and she was counseled regarding the need for regular gynecological follow-ups. All postpartum check-ups conducted so far went smoothly and showed no evidence of polypoid endometriosis recurrence or long-term fertility issues.

## 5. Discussion

To comprehensively analyze the existing literature on polypoid endometriosis, we systematically selected articles from the Web of Science and PubMed databases utilizing the following keywords: polypoid endometriosis, pregnancy, diagnosis, malignancy, imaging, treatment, surgical intervention. In order to refine our selection of pertinent articles, we established predefined inclusion criteria that encompassed studies involving human participants, research focusing on the diagnosis, treatment, or epidemiology of polypoid endometriosis, and investigations detailing surgical approaches in cases of ovarian neoplasm in pregnant women. Only articles published in English, with accessible full texts and released between 2004 and 2025, were deemed suitable for inclusion in this review. However, it should be mentioned that there are a few studies specifically focused on polypoid endometriosis, with only around 20 publications available. These publications include various types such as case reports, imaging studies, and literature reviews.

Polypoid endometriosis that mimics a neoplasm is a rare condition, particularly in young pregnant women. A review of the specialized literature reveals no documented cases specifically addressing polypoid endometriosis with neoplasm-like features in this demographic. Most comprehensive studies, including a well-known analysis of 24 cases of polypoid endometriosis by Parker R., focused on age groups ranging from 23 to 78 years, without highlighting any pregnant patients.

Other relevant reports include a case of a 34-year-old non-pregnant woman under the age of 40 who had vaginal polypoid masses successfully treated conservatively [26]. Additionally, there is a report by Lambrechts [27] concerning polypoid endometriosis of the bladder during pregnancy that mimicked urachal carcinoma. This case featured a vesicouterine polypoid mass that, while clinically and radiologically suspected to be malignant, was ultimately confirmed through histology to be pseudotumoral polypoid endometriosis of the bladder. Mahiou, K., described polypoid endometriosis as an exceptional subtype of endometriosis that can mimic aggressive pelvic cancer. Tsai C., also presented polypoid endometriosis as a rare entity that mimics ovarian cancer. Various singular cases have involved young or postmenopausal women, some undergoing hormonal treatments, yet none have been documented in pregnant patients. Schwab described a case report and literature review concerning polypoid endometriosis in a postmenopausal woman treated with tamoxifen [28]. Another study reported by Kim, J. Y., is a case of a young woman with a history of hormone treatment who was suspected of having an ovarian malignancy with metastases. Finally, Ling presented a case of polypoid endometriosis affecting the rectum and vagina in an adolescent [29].

Recent articles have discussed cases of polypoid endometriosis that mimic neoplasms in young pregnant women. A recent case report and review of the literature describing a case of bilateral ovarian tumor-like lesions detected during pregnancy was recently published by Zhang, Y.R. [30]. A study published by Maggiore, U., L.R., [31] emphasizes the challenges associated with diagnosing endometriotic lesions during pregnancy and explores treatment options for decidualized endometriosis in the context of imaging and symptomatology. Additionally, it outlines the potential acute complications of pregnancy related to pre-existing endometriosis and evaluates possible treatments for these complications. Finally, it assesses the impact of endometriosis on pregnancy outcomes and proposes mechanisms that may explain these underlying connections.

Distinguishing polypoid endometriosis from ovarian neoplasms during pregnancy presents a nuanced and intricate challenge that remains underexplored mainly in the medical literature. While comprehensive studies dedicated solely to this differentiation in the context of pregnancy are scarce, a handful of insightful case reports and reviews exist, offering a wealth of invaluable information. These resources illustrate the complexities of the condition, shedding light on the subtle differences that can profoundly impact diagnosis and treatment. Thus, a case report discusses how decidualized endometriomas can resemble malignant ovarian tumors during pregnancy, highlighting the diagnostic challenges and the importance of careful evaluation to avoid unnecessary interventions [32,33]. There are also studies that highlight the imaging features of polypoid endometriosis that can lead to confusion with malignant tumors, underscoring the need for awareness of this condition to prevent misdiagnosis [34,35].

The available reports indicate that, although focused studies are scarce, the current body of literature acknowledges the diagnostic difficulties associated with polypoid endometriosis during pregnancy [36,37].

In the case presented in our study, differentiating between polypoid endometriosis and a neoplastic growth on the ovaries in a pregnant patient proved to be challenging due to various physiological and pathological factors. Fortunately, the histopathological tests conducted successfully ruled out the presence of a malignant process, leading to a positive outcome in this instance.

It is crucial to underscore that the clinical presentations of cases resembling both ovarian neoplasms and polypoid endometriosis can share alarming similarities. Symptoms such as pelvic pain may arise from factors like cyst rupture, torsion, or pressure, leading to significant discomfort. The enlargement of an ovarian mass can easily be mistaken for a malignant tumor, creating a challenging scenario for both patients and healthcare providers. During pregnancy, the intricate hormonal landscape, characterized by elevated estrogen and progesterone levels, may spur the growth of endometriotic implants. This stimulation can result in these implants taking on grotesque transformations, mimicking the appearance of aggressive ovarian malignancies with solid components, cystic alterations, and irregular edges on imaging studies.

Additionally, some limitations of imaging methods must also be taken into account. Thus, ultrasound is used as the main diagnostic tool, but in pregnancy, ultrasound findings of polypoid endometriosis may mimic features seen in ovarian or endometrial cancer (e.g., borderline tumors or epithelial ovarian carcinoma). Also, information generated by Doppler ultrasound may show increased vascularity, but this feature can occur in both malignancy and endometriosis. Furthermore, it should be noted that fetal safety concerns are paramount, so the use of MRI to clarify the diagnosis is limited. These factors combined can exacerbate the risk of misdiagnosis in pregnant women, given the overlapping clinical features and the limited or delayed options for biopsy. In such precarious situations, the stakes are particularly high, warranting vigilant consideration and careful management.

In consequence, it should be emphasized that further research is needed to develop comprehensive guidelines for distinguishing between these conditions in pregnant patients.

## 6. Conclusions

The case of polypoid endometriosis mimicking a neoplasm in a young pregnant woman is a diagnostic and therapeutic conundrum due to its rarity and the critical need to distinguish a benign condition from a malignancy. Monitoring and prognosis are highly individualized, but the report of this case provides valuable information both on the pregnancy carried to term after surgical intervention and on the expected long-term outcomes, especially about the mother’s reproductive health.

Highlighting rare and intriguing cases contributes to increasing awareness of lesser-known conditions and may contribute to improving patient outcomes through early diagnosis and selection of appropriate intervention. Furthermore, by focusing on a rare and previously unreported case, the topic addressed emphasizes the importance of vigilance, adaptability, and continuous learning in the medical field. Thus, this review presents an invaluable opportunity to deepen our understanding of polypoid endometriosis in pregnant women, to enhance the diagnostic strategies employed for this complex condition.

Beyond the clinical implications, the psychological impact of a potential cancer diagnosis during pregnancy cannot be overlooked. The anxiety and emotional distress that can be generated by such a diagnostic suspicion can significantly affect a pregnant woman’s well-being, potentially resulting in impaired prenatal bonding and heightened concerns regarding future fertility and survival. By balancing maternal–fetal safety with appropriate medical or surgical interventions, clinicians can optimize both immediate and long-term reproductive health while minimizing unnecessary emotional distress for expectant mothers.

By refining diagnostic protocols and through the integration of precise diagnostic methodologies, interdisciplinary collaboration, and a patient-centered approach, the complexities associated with rare cases can be effectively managed.

## Data Availability

The data of study is available from corresponding authors upon request.
